# Enhanced MRI and MRI-Guided Interventional Procedures in Women with Asymptomatic Silicone-Injected Breasts

**DOI:** 10.1100/2012/549801

**Published:** 2012-03-12

**Authors:** Yun-Chung Cheung, Shin-Chih Chen, Yung-Feng Lo

**Affiliations:** ^1^Department of Medical Imaging and Intervention, Chang Gung Memorial Hospital, Linkou, Tao-Yuan 333, Taiwan; ^2^College of Medicine, Chang Gung University, Tao-Yuan 333, Taiwan; ^3^Department of Surgery, Chang Gung Memorial Hospital, Tao-Yuan 333, Taiwan

## Abstract

Asymptomatic women who have received silicone injection for breast augmentation have a risk of underestimating breast cancer by palpation, mammography, or breast sonography. Enhanced breast MRI is sensitive to display certain nonspecific enhanced lesions or suspicious lesions. Such nonspecific MRI-detected lesions could be managed by American College Radiology BI-RADS lexicon and selectively with MRI-guided techniques biopsy to prevent unnecessary surgery.

## 1. Introduction 

Liquid silicone injection was first used for breast augmentation during the 1950s and 1960s [1]. Although the US Food and Drug Administration (FDA) has never approved the use of silicone injection, it has been illicitly performed by both physicians and nonphysicians in the United States, Mexico, and Asia [2, 3]. In patients who have received direct silicone injection, various silicone-induced breast diseases, including local granulomatous reactions, mastitis, foreign body reaction, fibrosis, silicone migration, and autoimmune reactions, are common [1–6]. These reactive changes might induce hard, nodular breast masses or structural distortion mimicking neoplasm in certain cases. The masses containing silicone are often pathologically diagnosed as so-called silicone granulomas or siliconomas, which are difficult to differentiate from breast cancer by conventional mammography or sonography due to the generalized increased density of fibrosis and siliconoma.

Women who first received these silicone injections are now at the age when a high incidence of breast cancers occurs. In asymptomatic patients with silicone injection, however, breast tumor detection by mammography or sonography is controversial in terms of accuracy. They are thus facing the risk of misdiagnosis or delayed diagnosis. A breast mass becomes insensitive by palpation and becomes occult within a tensed to hardened breast. Enhanced magnetic resonance imaging (MRI) is believed to be an ideal imaging modality for further evaluation, but no reports exist concerning MRI findings in women with asymptomatic silicone-injected breasts.

 In this study, we reviewed our clinical data for enhanced MRI of women with asymptomatic silicone-injected breasts to analyze MRI findings and MRI-guided interventional procedures.

## 2. Patients and Methods

We retrospectively reviewed 33 consecutive patients aged 37–87 years (mean age, 59 years) with asymptomatic silicone-injected breasts who underwent breast MRI from 2008 to 2010 at our hospital. The results of mammography and breast sonography were inconclusive in all patients.

 For further evaluation, the patients were scheduled to undergo enhanced MRI of the breasts with a 1.5 T (HDx Twin; GE, Milwaukee, WI, USA) or 3 T (Tim Trio; Siemens, Erlangen, Germany) imager using a standard protocol and dynamic enhancement technique. The imaging protocols included axial T1-weighted imaging, axial and sagittal T2-weighted short *τ* inversion recovery (STIR) pulse sequences with fat and water saturation, and axial dynamic enhanced imaging on T1-weighted images with fat suppression (Siemens 3D turbo-FLASH pulse sequence: TR = 4 ms, TE = 1.68 ms, flip angle =20°, field of view 34 × 34 cm, postcontrast 8 cycles with 1 min/cycle or GE 3D VIBRANT pulse sequence: TR = 7.3 ms, TE = 3.3 ms, flip angle = 10°, field of view 34 × 34 cm, postcontrast 4 cycles with 2 min/cycle). We routinely used axial images for dynamic enhancement evaluation because we could compare the enhancement of both breasts at the same time, as well as document the distribution of the enhanced lesions, whether symmetric or asymmetric. Dynamic enhancement was performed by a bolus injection of 0.1 mmoL/kg gadopentetate dimeglumine (Magnevist; Bayer Schering Pharma, Berlin, Germany) via an intravenous catheter preset at the cubital region. MRI then continuously captured the kinetic enhancement of both breasts for 8 min. The postenhanced acquisition data were reconstructed by maximum intensity projection and subtraction images for evaluation.

**Figure 1:**
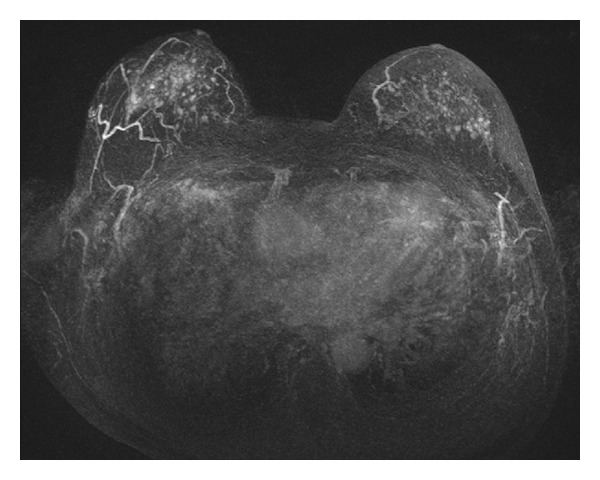
Axial 3D maximal intensity projection of enhanced breast MRI with fat suppression showed numerous foci in bilateral breasts.

**Figure 2:**
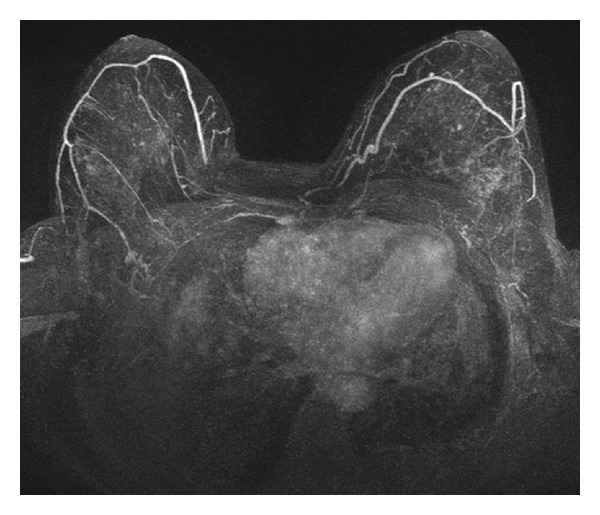
Axial 3D maximal intensity projection of enhanced breast MRI with fat suppression shows diffuse nonmass infiltrating enhancement symmetrically in bilateral breast parenchyma.

 The MRI examinations were reported by an experienced radiologist (>10 years experience). The examination results were subdivided as follows: normal without enhanced lesions (BI-RADS 1), suspicious benign enhanced lesions (BI-RADS 2 or 3), or suspicious malignant enhanced lesions (BI-RADS 4 or 5). The classification was based on the morphology of enhanced lesions according to the Breast Imaging Reporting and Data System (BI-RADS) lexicon of the American College of Radiology (ACR). Foci or nodules ≤5 mm (Figure 1), or multiple diffuse nonmass enhancements (Figure 2), either unilateral or bilateral, were suspicious of benign proliferative changes such as adenosis or fibrocystic breast disease. For an enhanced mass (>5 mm), smooth outlines were considered to be benign. Otherwise, MRI guidance procedures for biopsy were essentially recommended when the masses were lobulated or had an irregular outline or the nonmass enhancements were focal, linear, ductal, segmental, regional, or had a clumped appearance.

 MRI guidance procedures were first introduced at our hospital in 2008. All the MRI guidance procedures were performed by the same radiologist who is specialized in breast imaging, including mammography, sonography, and biopsy with stereotactic, sonographic, or MRI guidance procedures. After obtaining informed consent from the patients, MRI was performed by a 1.5 T MRI machine (HDx Twin; GE) with the patients in the prone position. The biopsied breast was immobilized by compression with a grid plate. After localization of the enhanced biopsy target, MRI-guided, vacuum-assisted, 10-gauge core needle biopsy (MRI-VAB) (Bard, Vacora, Covington, CA, USA), or preoperative needle localization with an MRI-compatible titanium hooked guide wire (Bard, USA) was performed for histopathological evaluation.

## 3. Results

Among the 33 asymptomatic patients, 16 had enhanced lesions, which were manifest as multiple enhanced foci in eight (Figure 1), enhanced masses in five, bilateral diffuse non-mass enhancement in two (Figure 2), and segmental nonmass enhancement in one.

**Figure 3:**
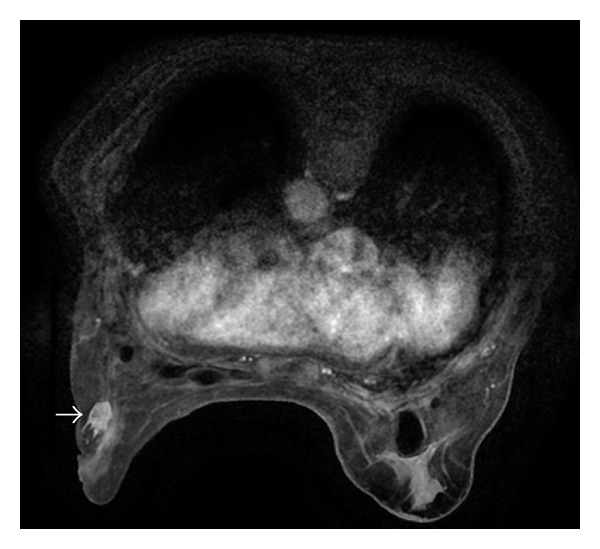
Axial fat-suppressed postcontrast breast MRI shows a 3 cm enhanced irregular mass with focal irregular (white arrow) in the left breast under compression before MRI-guided biopsy. It was diagnosed as ILC by the biopsy.

**Figure 4:**
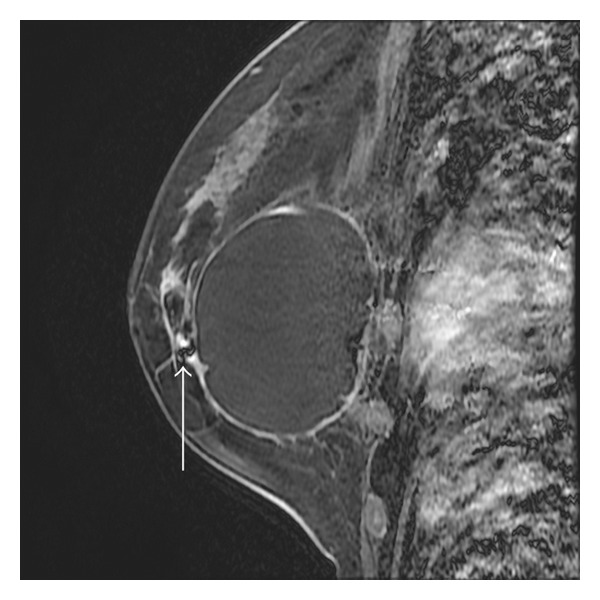
Sagittal postcontrast breast MRI of an augmented breast with silicone injection and mammoplastic bag placement showed a 0.8 cm lobular mass beneath the augmented mammoplasty bag, with placement of an MRI-compatible guide wire (white arrow) for excision localization.

**Figure 5:**
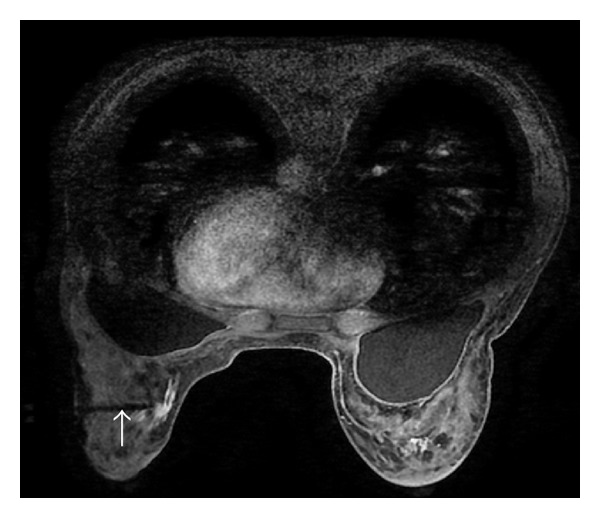
Axial postcontrast breast MRI in a patient who received silicone injection and mammoplastic bag placement shows a segmental enhanced lesion in left breast with a biopsy coaxial needle (white arrow) placed nearby. The biopsy diagnosis was adenosis.

 The five patients who displayed enhanced masses and the one with segmental nonmass enhancement were suggested for MRI-guided biopsy. However, only four women agreed to receive MRI-guided interventional procedures, which included vacuum-assisted core needle biopsy in three and preoperative needle localization in one (Table 1). The MRI appearance of biopsied lesions was lobular masses in two cases, and irregular mass and segmental nonmass enhancement in one each. With regard to the MRI lexicon, two irregular or lobular masses with enhanced intratumoral septa were suggestive of possible malignancies. The irregular mass was diagnosed as invasive lobular cancer (Figure 3) and the lobular mass as fibroadenoma. Another lobular mass showed benign features, including a smooth outline, homogeneous mild enhancement, and a continuously increasing intensity time curve. We decided to perform MRI-guided needle localization for excision biopsy due to the proximity of the lesion to the mammoplastic bag. The diagnosis turned out to be fibroadenoma (Figure 4). The segmental nonmass, enhanced lesion was shown pathologically by MRI-VAB to be adenosis (Figure 5). Among these four suspicious lesions, three showed moderate or rapid enhancement, either with washout or a plateau curve. Only one case was found to be cancerous. No cancer was discovered in the two women who refused MRI-guided procedures during 2 and 3 years of followup. Among the MRI-guided cases, three patients had a clinical history relating to cancer. The clinical information, MRI appearance, and dynamic enhancement patterns are described in Table 1.

No malignancy was discovered in this series with at least 1-year followup. Overall, only one of the 33 asymptomatic patients was histologically diagnosed with breast cancer, but this accounted for 25 percent (1/4 patients) among those who received MRI-guided interventional procedures.

## 4. Discussion

Direct injection of liquid silicone has been used in the past for breast augmentation. Silicone-related breast disease frequently leads to hard or tender breasts that make palpating breast masses difficult. Most of the cancers in silicone-injected breasts were discovered and evaluated because of palpation. A series of 16 women with lumpy silicone-injected breasts underwent enhanced MRI, and eight lumps were histologically diagnosed as invasive ductal carcinoma in three patients, silicone-induced mastitis in two, foreign body reaction in two, and silicone granuloma in one [7]. In another series, five of six surgically proven breast cancers were associated with palpable masses [8].

 Even though no evidence exists of silicone injection being related to the occurrence of breast cancer, the reactive changes in breasts can lead to difficulty in diagnosing breast cancer by conventional mammography or sonography, particularly at an early stage. Enhancement of breast lesions secondary to contrast medium leakage into the interstitial spaces of the lesions has resulted in MRI becoming the best or most sensitive imaging modality for identifying the lesions, despite the structural distortion in the parenchymal background. Enhanced MRI is thus believed to be the optimal technique for detecting lesions in difficult cases with silicone-injected breasts.

 In patients with liquid silicone injection, mammography usually demonstrates dense breast tissue with parenchymal distortion and opacity secondary to foreign body reaction or fibrosis, as well as the presence of silicone deposits randomly disseminated within the breasts, axillary regions, or elsewhere. The typical diffuse involvement of the breast by silicone granulomas or fibrosis can obscure coexisting neoplasms [9]. Similarly, the strong reflection, refraction, reverberation, and attenuation of sonographic beams by an overwhelming presence of silicone-induced fibrosis or silicone granuloma can lead to diffuse acoustic shadowing, which substantially hinders observation. Loss of definition of the posterior parenchyma has been reported as a common feature that is associated with sonography [9]. The usefulness of these two conventional imaging examinations is thus limited.

 Enhanced MRI has been documented as a better modality for patients with liquid silicone injection [7, 8, 10]. The consecutive high-resolution tomographic imaging of breast MRI resolves the superimposition of breast lesions under a heterogenous mammographic background. With the addition of the enhanced technique, fast MRI greatly improves spatial and temporal resolution of imaging, thus enabling detection of angiogenic breast lesions or evaluation of lesion morphology and extension.

 To standardize breast MRI reporting, the ACR established the BI-RADS MRI lexicon in 1998 [11], particularly for describing two major categories of morphology and enhancement kinetics. Morphologically, lesions can be separated into focus/foci (≤5 mm), mass (>5 mm), and nonmass enhancements. A focus is an enhanced spot ≤5 mm. As a result of its small size, it cannot be well characterized morphologically. Multiple foci may cause a spurious result for analysis of the enhancement kinetic curve because of the volume-averaging effect with surrounding normal tissue in the selected region of interest [11]. A focus is usually due to a benign lesion, such as focal fibrocystic change [12], papilloma, small fibroadenoma, or intramammary lymph node, although, rarely, it may also represent focal ductal carcinoma *in situ *(DCIS) or small invasive cancer [13].

 A mass is a three-dimensional space-occupying lesion and is characterized by shape (round, oval, lobulated, irregular), margin (smooth, irregular, spiculated), and internal mass enhancement characteristics (homogeneous, heterogeneous, rim enhancement, dark internal septations, enhancing internal septations, and central enhancement).

 Nonmass enhancement is characterized by distribution pattern (focal, linear, ductal, segmental, regional, multiple regions, and diffuse). Whether the enhancement distribution is symmetric or asymmetric between the breasts should also be determined. Multiple regions of enhancement or diffuse enhancements are more characteristic of benign proliferative changes. Although multicentric carcinoma, such as intraductal carcinoma or invasive lobular carcinoma (ILC), may exhibit a similar appearance, these findings are nearly always unilateral [13–15], which is why we did not encourage our eight patients with symmetrical, numerous enhanced foci in both breasts to undergo biopsy.

 Among the various shapes, irregular shape is the highest interobserver agreement for cancer, and lobular shape is suggestive for fibroadenoma [16]. However, a lobular mass without septations, a mass with enhancing septations, or moderate-to-marked, heterogeneous mass enhancement, with washout kinetics, is highly suggestive of malignancy but not specific for certain types of cancer [17]. For our two breast masses with features that were suggestive of possible malignancy, the irregular mass and lobular mass with enhanced intratumoral septa were diagnosed as cancer and fibroadenoma, respectively, by MRI-VAB.

 Liberman et al. have concluded that biopsy is rarely necessary for lesions smaller than 5 mm because of their low (3 percent) likelihood of cancer [12]. Nevertheless, the management of a focal lesion should depend on other findings in the same or opposite breast (such as symmetry) and corresponding findings from mammography or ultrasound, as well as the risk status of the patient [13, 18]. Enhanced nonmass lesions are neither a mass nor a blood vessel. The enhancement pattern is distinct from normal surrounding breast parenchyma without space-occupying effects. Benign or malignant lesions could present as non-mass-like enhancement, such as DCIS, ILC, mastopathic changes (focal adenosis), fibrocystic changes due to hormonal stimulation, or inflammatory changes [13, 18].

 Breast tumor intensity time curves are supposed to allow us to distinguish malignancy from benignity, based on different neoangiogenesis formations [19, 20]. Tumor neoangiogenesis or vascular permeability disruption by benign disease could induce contrast medium leakage into the interstitial spaces. However, the quantitative results vary depending on analysis methods and acquisition parameters. In addition to the interpretation of early enhancement, the late phases of dynamic enhancement curves with washout, plateau, or persistent increases are often used in clinical practice. Malignant tumors usually have rapid or strong enhancement, and benign lesions mostly show slow continuously increasing enhancement [21]. Unfortunately, benign or malignant lesions may reveal overlapping morphological features or dynamic enhanced patterns [22, 23]. The sensitivity of enhanced MRI for breast cancer has been reported as high (91 percent), but it has low specificity (37 percent) and diagnostic accuracy (58 percent) [24]. However, with addition of the patterns of dynamic signal intensity time curves, the diagnostic accuracy for cancer increases to 86 percent [24]. Unfortunately, this improvement cannot guarantee the diagnosis of malignancy from other benign angiogenic processes [24].

 Due to the limited specificity of enhanced MRI, histological diagnosis by percutaneous core needle biopsy may help to avoid unnecessary surgery on benign MRI-detected lesions. MRI-guided core needle biopsy was first introduced in 1997 by Heywang-Köbrunner et al., who also documented that a vacuum-assisted core needle has much better performance for obtaining large tissue volumes and hence reduces sampling error [25]. Many authors have recommended second-look sonography for MRI-detected breast lesions that are suspicious or highly suggestive of malignancy. If a sonographic correlate for the MRI-detected lesion is confidently identified, biopsy is usually performed under sonographic guidance [26]. In cases without sonographic correlates or only vague sonographic findings, MRI-guided biopsy needs to be considered [27]. However, we prefer to use the same imaging modality that confidentially revealed the suspicious lesions as the guiding tool. In contrast with our findings for silicone-injected breasts, subsequent management with MRI-guided interventional procedures for biopsy is indicated because of poor demonstration by sonography.

 A European multicenter study has reported no false-negative results among 517 successful MRI-VAB procedures [28]. The current results show that MRI-VAB is a reliable method that can obviate unnecessary surgery. However, MRI-VAB may not be feasible in some patients who cannot tolerate the procedures for various reasons, such as insufficient breast thickness or target lesions close to the chest wall. In addition, in our biopsy case 4, the target lesion was located beneath the mammoplastic bag, which resulted in us performing preoperative wire localization rather than MRI-VAB. Nevertheless, these two procedures are technically similar and familiar to the performer.

## 5. Conclusions

From our small number of cases, we could not calculate the accuracy of MRI examinations or MRI-guided procedures for cancer diagnosis due to a lack of gold standard surgery. We understood that enhanced breast MRI would display certain enhanced lesions in such difficult cases; however, we selected suspicious lesions that required biopsy based on the ACR BI-RADS lexicon. In conclusion, enhanced breast MRI examination with MRI-guided interventional procedures is advised to prevent unnecessary surgery of silicone-injected breasts.

## Figures and Tables

**Table 1:** Summary of MRI-guided interventional procedures in four asymptomatic silicone-injected breasts.

Age (yr)	Right/left	Clinical history	MRI features	Enhancement curve	Procedure	Biopsy diagnosis
87	Left	Unknown-origin bone metastasis	3 cm irregular mass	Rapidly increasing curve with washout	Vacuum-assisted core needle biopsy	Invasive lobular cancer
54	Left	Breast mass accidentally discovered during staging of diagnosed colon cancer by CT	1.2 cm lobular mass with enhanced intratumoral septa	Moderately increasing curve with plateau	Vacuum-assisted core needle biopsy	Fibroadenoma
42	Left	Incidental discovered in health examination	1.5 cm segmental nonmass enhancement	Rapidly increasing curve with plateau	Vacuum-assisted core needle biopsy	Adenosis
51	Right	Left breast cancer after mastectomy 3 years before	0.8 cm lobular mass	Continuously slowly increasing curve	Wire localization	Fibroadenoma
